# Recyclable Photopolymers for Sustainable 3D Printing

**DOI:** 10.1021/polymscitech.5c00144

**Published:** 2026-03-03

**Authors:** Qirui Wu, Youyi Sun, Si Wu

**Affiliations:** † School of Materials Science and Engineering, 66291 North University of China, Taiyuan 030051, China; ‡ Hefei National Research Center for Physical Sciences at the Microscale, Anhui Key Laboratory of Optoelectronic Science and Technology, Department of Polymer Science and Engineering, 12652 University of Science and Technology of China, Hefei 230026, China

**Keywords:** 3D printing, recyclability, depolymerization, reprocessing, noncovalent bond

## Abstract

The use of photopolymers for 3D printing facilitates high-precision fabrication of geometrically complex structures, offering exceptional dimensional accuracy and rapid curing capabilities that position it as a cornerstone of modern additive manufacturing. However, conventional photopolymers form permanently crosslinked networks, which are resistant to recycling. This inherent limitation generates persistent waste streams that are fundamentally incompatible with circular economy principles. Consequently, the development of recyclable photopolymers that maintain printability while enabling closed-loop material recovery represents a critical Frontier for sustainable manufacturing. Advancing these materials will remain essential for reconciling technological progress with environmental stewardship in the foreseeable future. This review examines recent breakthroughs in recyclable photopolymer systems for 3D printing. First, mainstream photopolymerization techniques compatible with recyclable materials are outlined, followed by an elucidation of core design strategies incorporating chemical depolymerization, thermo-mechanical reprocessing, and noncovalent interactions. Fundamental recycling mechanisms are detailed alongside performance modulation methodologies. Furthermore, emerging applications in soft robotics, wearable devices, and bioelectronic devices where recyclable photopolymers enable multifunctional devices are highlighted. Finally, persistent challenges regarding network durability and recycling efficiency are addressed, and future research directions toward truly sustainable 3D printing are proposed.

## Graphical abstract

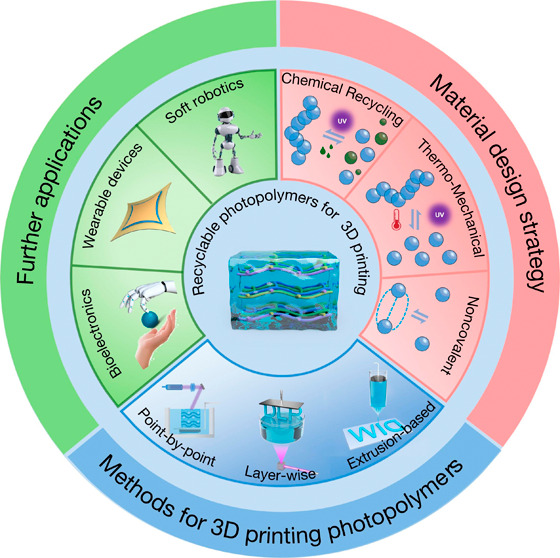

## 1 Introduction

Three-dimensional (3D) printing, serving as the core driver of additive manufacturing, has fundamentally transformed the limitations inherent in traditional subtractive techniques through its innovative layer-by-layer material deposition approach.^1–12^ This technology delivers unparalleled design freedom and manufacturing flexibility across diverse sectors including aerospace, biomedicine, electronics, automotive engineering, and consumer goods, significantly advancing the efficient fabrication of personalized, customized, and geometrically intricate structures.^13–23^ Among various 3D printing technologies, photopolymer-based processes occupy a pivotal position in high-precision prototyping and direct manufacturing of functional components. This prominence stems from their exceptional dimensional accuracy (reaching micrometer scale), superior surface finish, and rapid curing capabilities.^24–27^ The fundamental operating principle involves utilizing ultraviolet or visible light as an energy source to activate photoinitiators within liquid photopolymer resins.^28–30^ These initiators generate reactive species that subsequently drive monomers or oligomers to undergo chain polymerization or crosslinking reactions. This photochemical mechanism enables the rapid transformation from liquid precursors into solid 3D structures.^31–33^ However, the rapid advancement and widespread adoption of 3D printing technologies have increasingly revealed significant environmental concerns. Particularly problematic are conventional photopolymers, which typically form highly crosslinked networks upon curing.^34–36^ These cured materials exhibit inherent chemical inertness and structural stability, rendering them resistant to effective degradation or recycling through conventional physical or chemical methods. This limitation not only perpetuates the waste of valuable resources and exacerbates dependence on fossil fuels but also generates substantial volumes of persistent 3D printed waste. Such waste streams pose considerable ecological threats while actively hindering the development of environmentally sustainable 3D printing.^37–39^ This trajectory fundamentally contradicts global imperatives for carbon neutrality and circular economy principles.

Against this backdrop, developing recyclable photopolymers that simultaneously deliver excellent printability and efficient recyclability has emerged as a critical scientific challenge and Frontier research focus at the intersection of materials science and additive manufacturing.^40–43^ These next-generation materials must retain the superior photocuring characteristics of conventional photoresins, such as appropriate viscosity, rapid photoresponse kinetics, and tunable crosslinking density, alongside their desirable printing fidelity and mechanical performance.^44–46^ This is achieved by strategically incorporating dynamic or reversible bonds within the polymer networks or designing controllable polymerization-depolymerization pathways to enable material reprocessing. These dynamic motifs confer structural integrity and functional stability to printed 3D objects during their service life, while allowing network dissociation, reconfiguration, or chain depolymerization under specific stimuli (e.g., heat, light, chemical agents, mechanical force, or enzymes) upon reaching end-of-life or requiring disposal.^47–51^ This inherent responsiveness facilitates efficient material recovery, closed-loop recycling, or environmentally benign degradation. Consequently, advancing the fundamental research and technological development of recyclable photopolymer 3D printing offers substantial benefits.^52,53^ This approach not only mitigates polymer waste generation at its source, significantly reducing the environmental footprint of the 3D printing industry and alleviating mounting ecological pressures, but also can improve material utilization through the recycling of resources and reduce production costs, which is in line with the core requirements of sustainable development. Beyond these environmental and economic advantages, such materials establish a novel platform for fabricating advanced 3D printed devices endowed with integrated smart responsiveness and multifunctionality.^54–58^ This capability holds significant promise for unlocking novel applications in emerging fields such as soft robotics, wearable devices, and bioelectronic devices.

Herein, this review consolidates the latest research advances in recyclable photopolymer 3D printing technologies. It should be noted that biodegradable photopolymers represent another important approach toward sustainable 3D printing. Some biodegradable polymers, particularly those synthesized via ring-opening polymerization of cyclic monomers, can also exhibit recyclability depending on their ceiling temperature, enabling both end-of-life biodegradation and closed-loop chemical recycling pathways. While biodegradable photopolymers constitute a significant research area in their own right, this review focuses specifically on recyclable photopolymers designed for circular material flows, where the primary end-of-life strategy involves material recovery and reuse rather than environmental degradation.^59–61^ We begin by outlining mainstream photopolymerization-based 3D printing techniques and their compatibility with recyclable materials (Figure 1). Subsequently, we focus on elucidating material design strategies and core recycling mechanisms for recyclable photopolymers, encompassing chemical recycling, thermo-mechanical recycling, and noncovalent interactions. Building upon this foundation, we discuss performance modulation approaches and explore the application potential of these materials in emerging fields such as soft robotics, wearable devices, and bioelectronic interfaces. Finally, we provide an analysis of the major challenges and future research directions currently facing this field.

**Figure 1:**
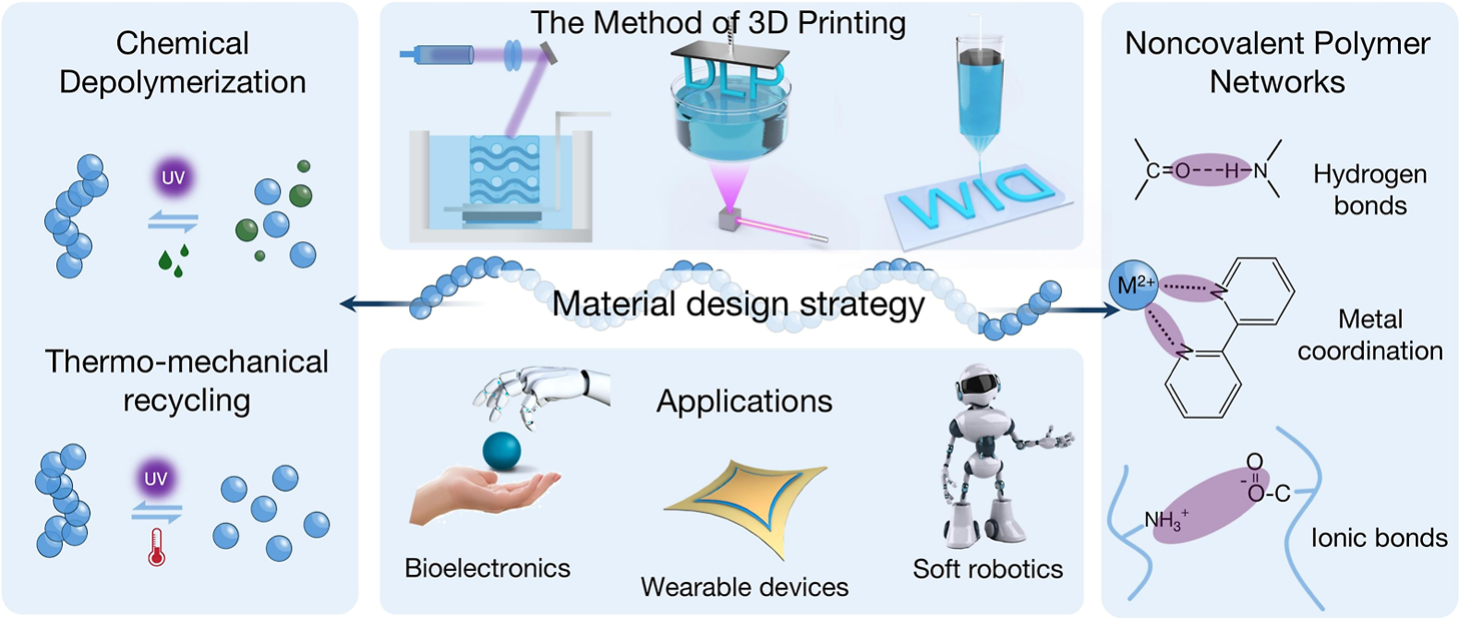
3D printing method, material design strategy, and application schematic of recyclable photopolymer.

## 2 Methods for 3D Printing Using Photopolymers

3D printing constructs objects via additive manufacturing, utilizing computer-controlled systems to replicate computer-aided design (CAD) models. These CAD models are digitally sliced into discrete layers that guide the printer in building three-dimensional structures (Figure 2).^62–65^ Within photopolymerization-based 3D printing, various techniques can be systematically classified according to their spatial curing modes-that is, how light energy is delivered to selectively solidify the photosensitive resin. This classification encompasses point-by-point scanning methods, layer-wise projection methods, and extrusion-based photocuring approaches, each offering distinct advantages and constraints for processing recyclable photopolymer systems.

**Figure 2:**
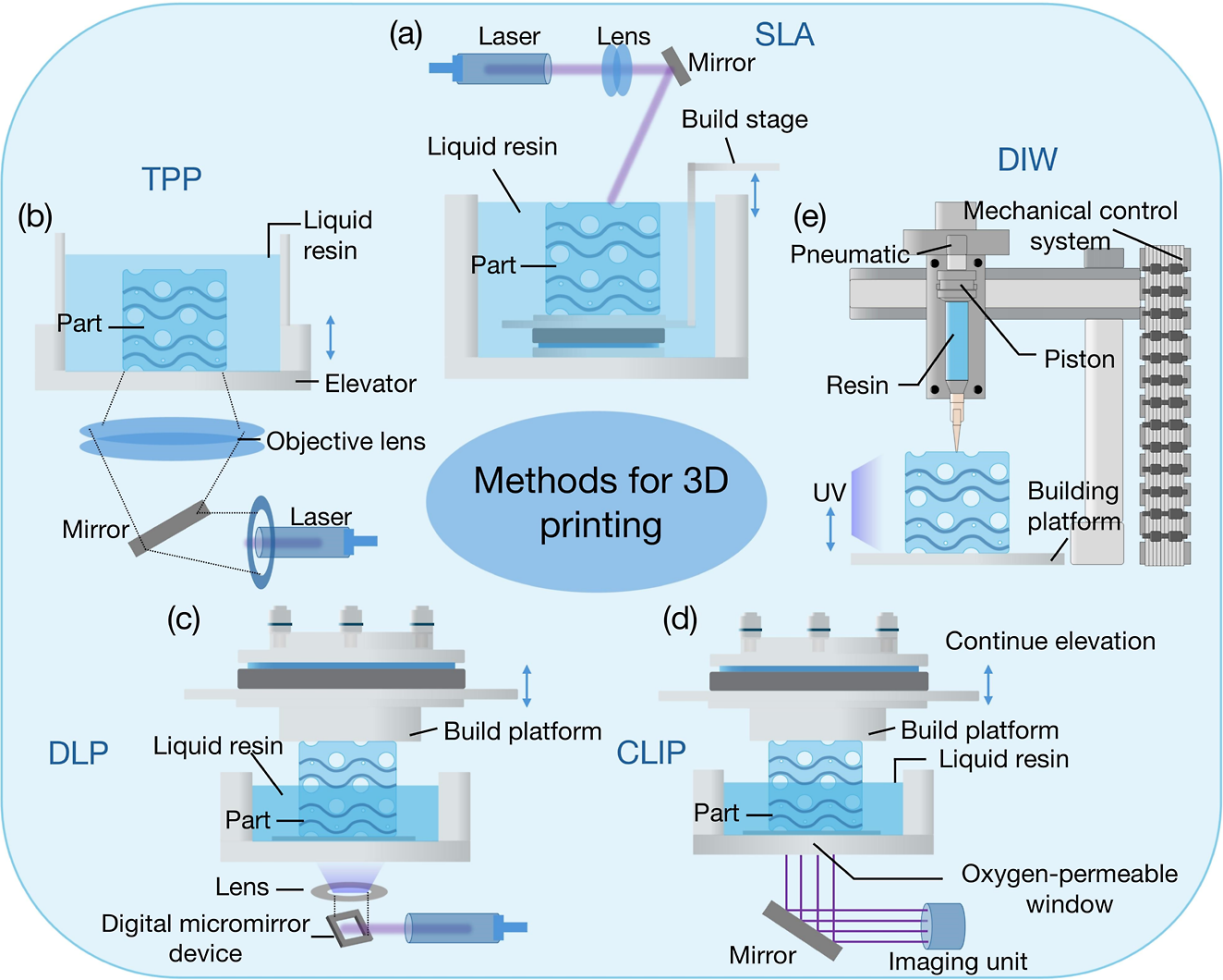
Schemes for 3D printing using photopolymers. (a) Stereolithography (SLA) printing. (b) Two-photon polymerization (TPP) printing. (c) Digital light processing (DLP) printing. (d) Continuous liquid interface production (CLIP) printing. (e) Direct ink writing (DIW) printing.

### 2.1 Point-By-Point Scanning Methods

Point-by-point scanning techniques employ a focused light source to trace and cure the resin along predefined paths, building each layer through sequential solidification of individual voxels or line segments. Stereolithography (SLA) represents the pioneering photopolymerization-based 3D printing technology. In SLA, a UV laser beam is precisely directed by galvanometer mirrors to scan across the surface of a liquid photopolymer resin, selectively curing material point-by-point according to the sliced pattern (Figure 2a).^66^ The build platform then repositions, and a fresh resin layer is applied for subsequent scanning. Modern SLA systems can be configured with the laser approaching either from above or below the resin vat, offering flexibility in addressing oxygen inhibition and part separation challenges. For recyclable photopolymer applications, SLA provides excellent control over local cure depth and crosslinking density, enabling precise placement of dynamic bond-containing domains within printed structures. Two-photon polymerization (TPP) achieves unparalleled spatial resolution by exploiting nonlinear optical absorption. When an ultrafast pulsed laser is tightly focused into a photosensitive resin, two photons are simultaneously absorbed only within a highly confined focal volume, initiating polymerization at the nanoscale (Figure 2d).^67^ This intrinsic three-dimensional confinement eliminates the need for layer-by-layer processing and enables direct writing of arbitrary 3D microstructures with sub-micrometer features. While TPP offers exceptional resolution for fabricating intricate recyclable microdevices, its relatively slow writing speed and specialized resin requirements currently limit throughput for larger-scale applications.

### 2.2 Layer-Wise Projection Methods

Layer-wise projection techniques cure entire two-dimensional cross sections simultaneously by projecting patterned light onto the resin surface, significantly accelerating build rates compared to point-scanning approaches. Digital light processing (DLP) utilizes a digital micromirror device (DMD) to project complete slice images onto the photopolymer resin, curing each layer in a single exposure (Figure 2b).^68^ The parallel nature of DLP enables rapid fabrication while maintaining good resolution determined by the projected pixel size. This technique has emerged as particularly favorable for recyclable photopolymer research due to its compatibility with a wide range of resin viscosities and its ability to achieve uniform crosslinking across each layer-a critical consideration for ensuring consistent dynamic bond distribution throughout printed parts. Continuous liquid interface production (CLIP) advances the DLP concept by introducing an oxygen-permeable window beneath the resin vat, creating a persistent liquid interface-termed the “dead zone”-that prevents adhesion between the curing part and the window (Figure 2c).^69^ This innovation enables continuous rather than stepwise vertical movement, dramatically increasing print speeds while producing parts with isotropic mechanical properties free from visible layer lines. The continuous nature of CLIP presents unique opportunities for recyclable photopolymers, as the reduced thermal cycling and mechanical stresses during printing may better preserve the integrity of dynamic covalent networks.

### 2.3 Extrusion-Based Photocuring Methods

Extrusion-based photocuring approaches decouple the material deposition step from the photopolymerization process, offering distinct advantages for processing high-viscosity or composite-laden recyclable formulations. Direct ink writing (DIW) with photocuring involves extruding a photocurable ink through a nozzle to form filamentary structures, followed by UV or visible light exposure to solidify the deposited material (Figure 2e).^28,31^ The sequential nature of this process-first shaping, then curing-accommodates highly viscous resins and enables incorporation of functional fillers that would otherwise impede light penetration in vat-based methods. For recyclable photopolymers, DIW facilitates the fabrication of multi-material structures with spatially programmed recyclability, where different dynamic chemistries can be selectively deposited in distinct regions. Furthermore, the ability to process paste-like materials opens pathways for incorporating recycled feedstock directly into new prints.

### 2.4 Technique Selection Considerations for Recyclable Photopolymers

The selection of an appropriate printing technique for recyclable photopolymers involves balancing multiple interrelated factors (Table 1). Point-scanning methods (SLA, TPP) offer superior control over local crosslinking but exhibit lower throughput. Layer-wise projection methods (DLP, CLIP) provide an optimal compromise between speed, resolution, and material flexibility, explaining their predominant use in current recyclable photopolymer research. Extrusion-based approaches uniquely accommodate high-viscosity formulations and multi-material architectures essential for functionally graded recyclable structures. Critically, the thermal and rheological requirements imposed by each technique directly influence the design of recyclable networks. High-resolution methods demand low-viscosity resins with rapid cure kinetics, potentially limiting the molecular weight of dynamic bond-containing oligomers. Conversely, extrusion methods tolerate-and often require-higher viscosities, enabling incorporation of larger dynamic motifs. These constraints necessitate tailored approaches to dynamic covalent chemistry selection, ensuring both printability during fabrication and efficient closed-loop reprocessability at end-of-life.

**Table 1:** Comparison of Photopolymerization-Based 3D Printing Techniques for Recyclable Photopolymers

methods	curing mode	resolution (μm)	build speed	viscosity	advantages
SLA	point scanning	25–100	several mm min^–1^	low–Medium	precise local control of dynamic bond density
TPP	point scanning	0.1–1	80 nm s^–1^ to 2 cm s^–1^	low	nanoscale recyclable microstructures
DLP	layer projection	1–100	25–1000 mm min^–1^	low–medium	uniform crosslinking; broad resin compatibility
CLIP	continuous projection	50–100	1000 mm h^–1^	low	isotropic properties; reduced layer interfaces
DIW	extrusion post-cure	100–500	1 mm s^–1^ to 10 cm s^–1^	high	multi-material; recycled feedstock integration

Beyond network design, photoinitiator selection represents another critical formulation consideration that varies with printing technique. Conventional photoinitiators such as 2,4,6-trimethylbenzoyl-diphenylphosphine oxide (TPO) and bis­(2,4,6-trimethylbenzoyl)-phenylphosphine oxide (BAPO) are widely employed due to their high initiation efficiency under UV–visible light (365–405 nm).^70,71^ However, general challenges including yellowing effects, migration to the surface, and cytotoxicity concerns must be addressed, particularly for biomedical applications.^72–74^ Different printing techniques impose distinct photoinitiator requirements. TPP necessitates specialized two-photon absorbing chromophores with high two-photon absorption cross sections.^75^ DLP and CLIP require photoinitiators matched to the projector wavelength (typically 385 or 405 nm) with appropriate molar absorptivity to balance cure depth and resolution, while DIW offers greater flexibility as photocuring occurs post-deposition.^70^

For recyclable photopolymers, additional considerations arise from the interplay between photoinitiators and recycling mechanisms. First, compatibility between photoinitiator chemistry and dynamic bond exchange or depolymerization processes must be ensured, as certain radical species or photolysis byproducts may interfere with catalyst activity or consume functional groups essential for recycling. Second, residual photoinitiators and their degradation products may accumulate across multiple printing-recycling cycles, potentially affecting optical properties and recycling efficiency. Third, for systems employing photocleavable moieties for light-triggered degradation, careful wavelength selection is critical to avoid spectral overlap between the printing wavelength and the degradation trigger. Emerging developments including visible-to-NIR light photoinitiators, bio-based photoinitiators derived from renewable feedstocks, and macromolecular photoinitiators with reduced migration tendency offer promising solutions to these challenges.^76–79^

## 3 Designing Recyclable Photopolymers for Sustainable 3D Printing

### 3.1 Design Principles and Classification

The development of recyclable photopolymers necessitates a fundamental departure from conventional permanently crosslinked networks toward architectures incorporating stimuli-responsive or reversible linkages.^80–82^ To provide a coherent framework for understanding the diverse strategies reported in the literature, it is essential to establish clear distinctions among the various recycling mechanisms, as these determine both the processing requirements and the quality of recovered materials. Recyclable photopolymer systems can be systematically classified into three principal categories based on their underlying recycling mechanisms.^83^ The first category, chemical recycling via depolymerization, encompasses systems wherein the polymer network undergoes controlled chain scission to regenerate monomers, oligomers, or other low-molecular- weight species that can be subsequently repolymerized. This approach offers the highest fidelity in material recovery, as the regenerated building blocks are chemically identical or equivalent to the original precursors, enabling theoretically unlimited recycling cycles without property degradation.^84–86^ The driving force for depolymerization may be thermal, chemical, or photochemical, depending on the specific bond chemistry employed. The second category, thermo-mechanical reprocessing via dynamic bond exchange, relies on network topology rearrangement rather than chain depolymerization. In these systems, commonly referred to as vitrimers or covalent adaptable networks (CANs), dynamic covalent bonds undergo associative or dissociative exchange reactions under elevated temperature or in the presence of catalysts, enabling stress relaxation and network reconfiguration while maintaining constant or near-constant crosslink density.^87–89^ This mechanism permits the reprocessing of crosslinked materials through conventional thermoplastic processing techniques such as hot pressing, extrusion, or injection molding, without requiring dissolution or depolymerization steps. The third category leverages noncovalent interactions, including hydrogen bonding, metal coordination, ionic association, and host–guest complexation, to construct reversible networks. The relatively low bond energies associated with noncovalent interactions enable network dissociation and reformation under mild conditions, offering energy-efficient recycling pathways. These systems may function independently or synergistically with covalent networks to achieve enhanced mechanical properties while retaining recyclability. Notably, these categories are non-mutually exclusive, and hybrid systems combining multiple mechanisms have been engineered to harness the complementary advantages of different approaches.^90,91^ The following sections provide detailed examinations of representative systems within each category, with emphasis on the molecular design principles, recycling methodologies, and structure–property relationships.

### 3.2 Chemical Recycling via Depolymerization

Chemical recycling through depolymerization represents the most thermodynamically complete approach to material recovery, as it regenerates the original monomeric or oligomeric building blocks from crosslinked networks. This strategy circumvents the progressive property degradation typically associated with mechanical recycling by resetting the material to its pristine molecular state. The feasibility of depolymerization is governed by the ceiling temperature (*T*_c_) of the polymer system, which defines the thermodynamic threshold above which depolymerization becomes entropically favored.^59,60^ For photopolymer systems, achieving efficient depolymerization requires the strategic incorporation of cleavable linkages within the network backbone or crosslinks.

Dynamic disulfide bonds have emerged as particularly attractive motifs for constructing depolymerizable photopolymer networks due to their susceptibility to thiol–disulfide exchange and reductive cleavage under mild conditions. Dove^92^ and colleagues developed a 3D printing resin achieving both efficient decomposition and high-resolution fabrication by replacing conventional (meth)­acrylates with dynamic cyclic disulfide species derived from lipoates (Figure 3a,b). This molecular design overcomes the inefficiencies previously encountered in photopolymers relying solely on internal dynamic covalent bonds for recycling. However, the progressive deterioration of mechanical properties caused by network damage and functional group depletion during recycling cycles remains a limitation. Addressing this challenge, Xie^80^ and colleagues developed a novel SLA 3D printing chemistry based on dithioacetal bond photopolymerization (Figure 3c,d). Their approach leverages selective dissociation of dithioacetal linkages to transform crosslinked networks into photo-reactive oligomers. Critically, this network-to-oligomer transformation is fully reversible, enabling the resin to retain its original mechanical robustness across multiple recycling cycles while achieving quantitative conversion. This reversibility represents a significant advance toward truly closed-loop 3D printing.

**Figure 3:**
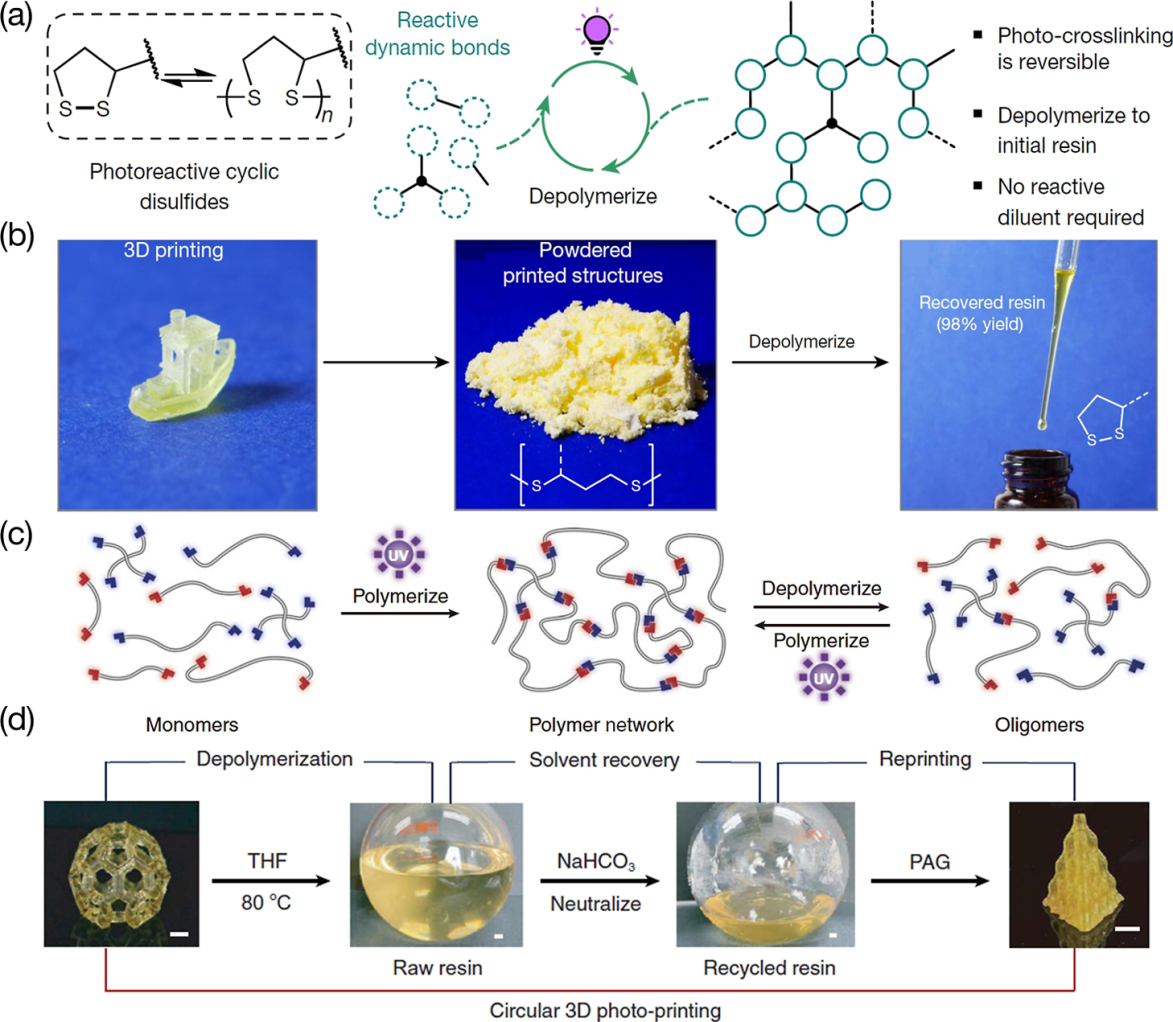
(a) Closed-loop recycling in 3D printing presents a method to achieve polymerization–depolymerization cycles of dynamic disulfide bonds that enables the creation of renewably sourced resins suitable for closed-loop chemical recycling. (b) 3D-printed complex part, photograph of powdered 3D-printed parts and photograph of recovered resin from depolymerized 3D-printed parts. Adopted with permission from ref 92. Copyright 2023, Springer Nature; (c) design of closed-loop recyclable photopolymer networks and molecular principle for the recyclable photopolymer networks. (d) Circular printing process. Scale bars, 5 mm. Adopted with permission from ref 80. Copyright 2025, AAAS.

Polymers synthesized via ring-opening polymerization (ROP) of cyclic monomers inherently possess depolymerizability when heated above their ceiling temperature. Yue^34^ et al. exploited this principle using acrylate-functionalized poly­(δ-valerolactone) (PVLA), synthesized via ring-opening transesterification polymerization of δ-valerolactone (VL), as a platform photoprecursor. Incorporation of reactive diluents transformed the non-printable PVLA into DLP-compatible inks with tunable properties suitable for sacrificial molds, soft actuators, and sensors. Crucially, the inherent depolymerizability of PVLA was preserved regardless of whether the printed product was thermoplastic or thermosetting. Direct bulk thermal decomposition achieved 93% recovery of the original VL monomer mass, demonstrating the potential of bio-renewable cyclic monomers as versatile platforms for circular DLP printing.

An alternative approach to achieving depolymerization employs photocleavable moieties that enable light-triggered network degradation. Wang^2^ et al. developed a multifunctional photoinitiator that photochemically controls both polymerization initiation and material degradation. Irradiation at 450 nm triggers crosslinking of monofunctional monomers, while 365 nm exposure promotes material degradation. This dual functionality was achieved by covalently linking bis­(acyl)­phosphane oxides and *o*-nitrobenzyl (ONB) moieties, followed by immobilization onto γ-cyclodextrin surfaces. The degraded material consists of linear polymers readily soluble in their corresponding monomers, facilitating straightforward resolidification with mechanical properties exceeding those of the original material.

### 3.3 Thermo-Mechanical Reprocessing via Dynamic Bond Exchange

In contrast to depolymerization strategies that regenerate monomeric species, thermo-mechanical reprocessing exploits dynamic covalent bond exchange reactions to enable network reconfiguration without chain scission. These systems, broadly classified as vitrimers or covalent adaptable networks (CANs), combine the dimensional stability and solvent resistance of thermosets with the reprocessability of thermoplastics. The recycling process typically involves comminuting printed objects into particulate feedstock followed by compression molding under elevated temperature and pressure. While this approach generally entails higher energy consumption than chemical recycling and carries risks of thermal degradation during prolonged exposure, it proceeds without requiring additional chemical agents or solvents.

Transesterification represents one of the most extensively studied dynamic exchange reactions for constructing reprocessable photopolymer networks. Zhang^93^ et al. employed 2-hydroxy-3-phenoxypropyl acrylate as the key monomer to construct a vitrimer resin system fabricated via DLP printing combined with a two-stage polymerization approach (Figure 4a). The first stage established a permanent covalent network through photopolymerization, while the second stage activated transesterification reactions between ester and hydroxyl groups at elevated temperatures, introducing dynamic character. Recycling was achieved through thermal treatment at 200 °C under 500 MPa pressure for 2 h. The material maintained performance over multiple cycles with only minor mechanical property reductions, offering a viable solution for recycling 3D printed thermosets. Building upon this foundation, Li^91^ et al. developed a UV-curable recycling system specifically designed for DLP printing of vitrimer waste. Their approach blends vitrimer powder with a specialized UV-curable solution containing both acrylate groups for photopolymerization and hydroxyl/ester groups for transesterification. This dual functionality enables direct fabrication of high-resolution 3D components from recycled feedstock, with subsequent thermal treatment activating bond exchange to enhance mechanical properties. This method bridges closed-loop thermoset recycling with sustainable 3D printing advancement (Figure 4b,c).

**Figure 4:**
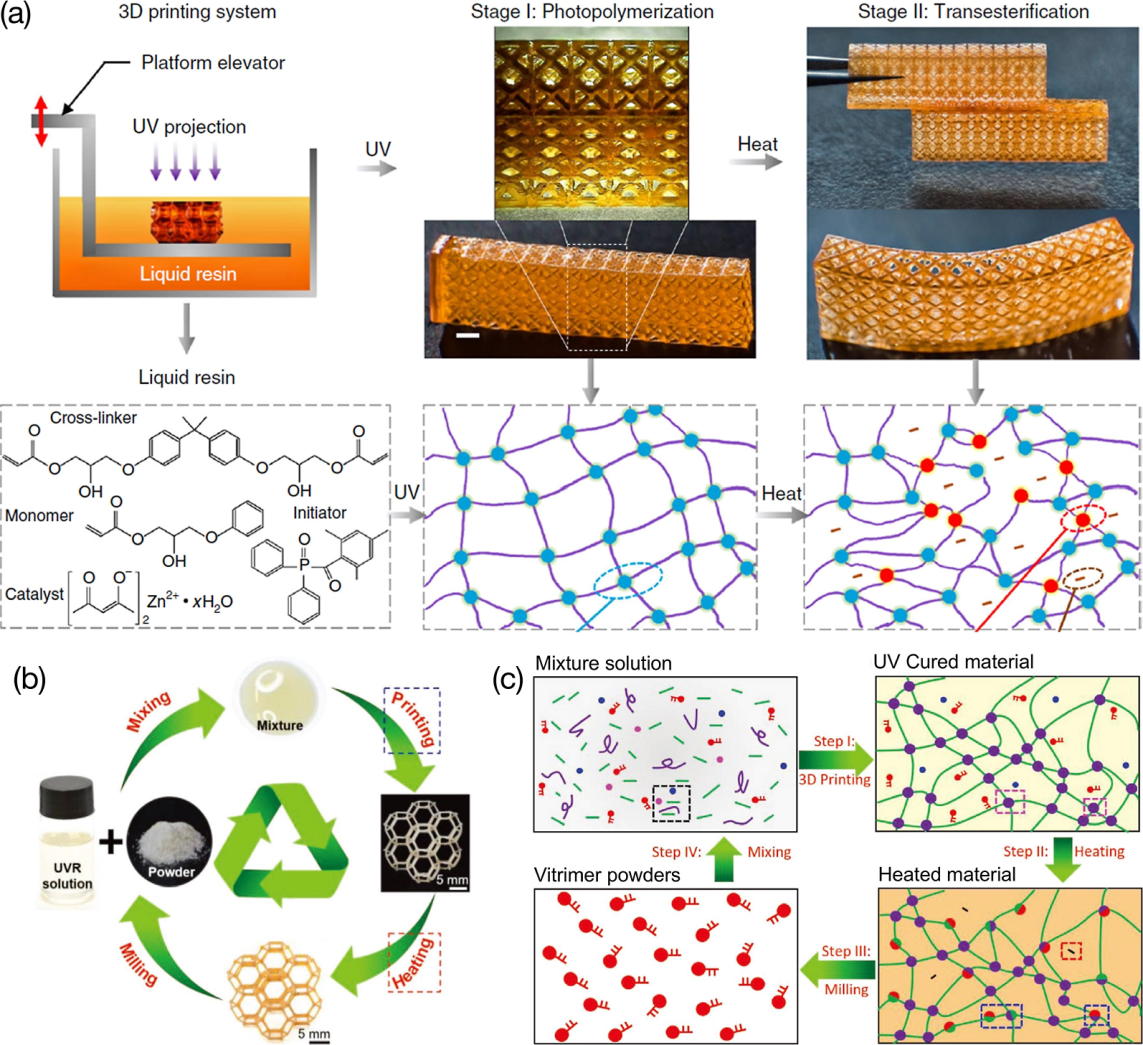
(a) General route of 3D printing high-resolution lattice structures with a UV curing-based 3D printing system using the reprocessable thermosetting polymer solution (stage I). Heating then imparting the reprocessability into the printed structures. Two separate printed lattice structures can be welded together, and a straight lattice structure can be programmed into a bent one (stage II). Adopted with permission from ref 93. Copyright 2018, Springer Nature; (b) illustration of a full cycle that reprocesses vitrimers into complex 3D geometry through DLP-based 3D printing. (c) Illustration shows the 3D printing and heat treatment process for recycling vitrimer. Purple dots: permanent covalent bonds; two-color dots: dynamic covalent bonds. Adopted with permission from ref 91. Copyright 2022, Wiley-VCH.

Achieving high recyclability while maintaining mechanical robustness remains a central challenge for vitrimer-based photopolymers. Gao^94^ et al. developed a robust and reprocessable acrylate vitrimer for stereolithography (SLA) 3D printing by combining exchangeable β-hydroxyl esters with sacrificial hydrogen bonds. The incorporation of acrylamide (up to 20 wt %) into the formulation significantly enhanced mechanical performance, achieving tensile strengths of approximately 40 MPa and Young’s moduli of 871 MPa while maintaining full reprocessability. The hydrogen-bond-integrated networks provided synergistic reinforcement without compromising the dynamic exchange capability of the ester linkages. This SLA compatible vitrimer system enabled the precise fabrication of recyclable 3D objects with complex architectures, demonstrating that point-by-point laser curing can achieve comparable dynamic network performance to layer-wise projection methods (Figure 5a–d).

**Figure 5:**
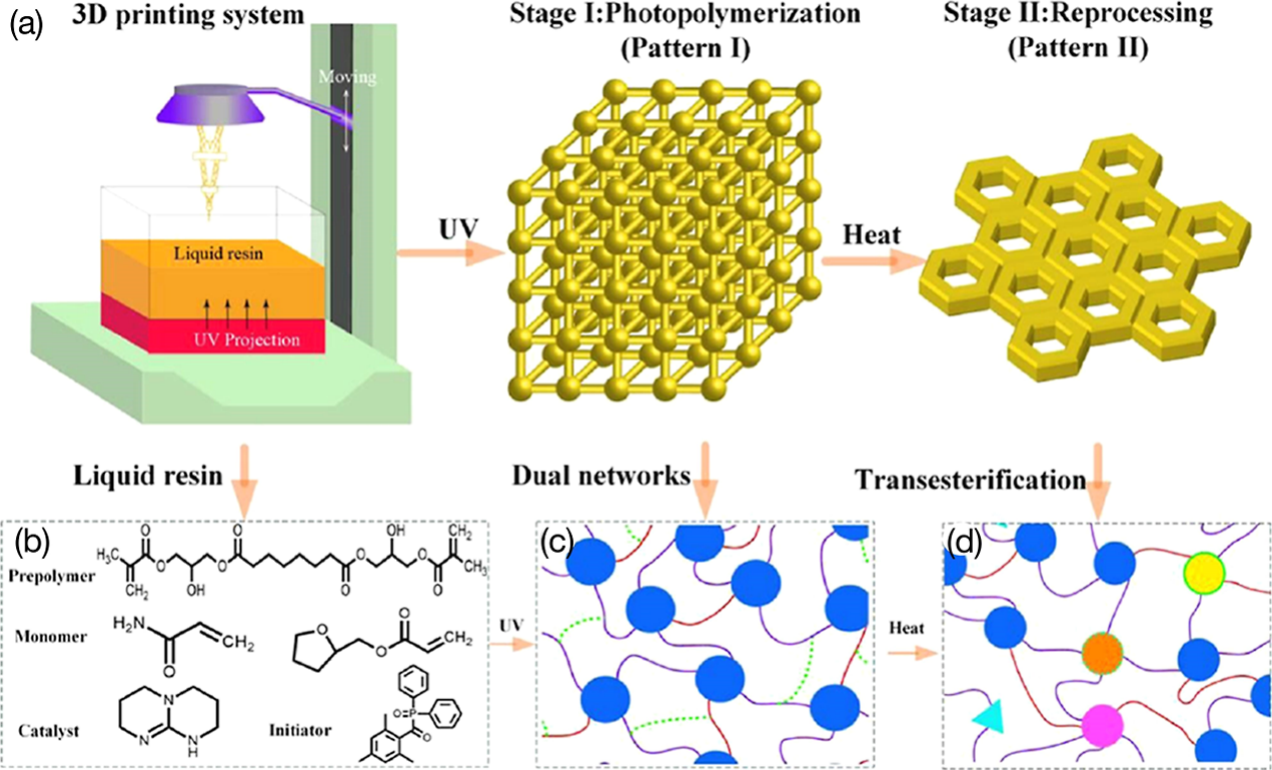
Three-dimensional (3D) printing mechanically robust and reprocessable acrylate vitrimers. (a) General path of a photocurable 3D printing system using acrylate vitrimers as 3D printing materials (stage I). Reprocessable vitrimers are achieved by heating and dissolving them, endowing the reprocessability of 3D printing materials by ester exchange reactions (stage II). (b) Chemical structures of prepolymer, monomer, catalyst, and initiator for synthesizing acrylate vitrimers. (c) Photopolymerization generates the permanent chemical cross-link dots (blue dots) and hydrogen bonds (green dotted line). (d) Thermal-activated transesterification results in the recombination of dynamic covalent bonds (purple, yellow, orange, and green dots). Adopted with permission from ref 94. Copyright 2024, American Chemical Society.

Extending vitrimer processing to extrusion-based techniques, Jehl^95^ et al. demonstrated a bio-based, UV-active, recyclable polybenzoxazine vitrimer fabricated via UV-assisted material extrusion. A ditelechelic benzoxazine monomer containing ester groups and aliphatic hydroxyl groups was synthesized from sustainable feedstocks. Partial methacrylation imparted UV-curability while preserving ester-hydroxyl pairs for transesterification-based dynamic exchanges. The optimized formulation enabled 3D printing followed by thermal post-curing, yielding vitrimer parts with *T*_α_ of 105 °C and compressive modulus of 810 MPa. Notably, ground 3D printed parts could be directly reused as viscosity modifiers in fresh resin formulations, establishing a practical closed-loop recycling strategy. This work demonstrates that extrusion-based photocuring can accommodate higher-viscosity vitrimer formulations while maintaining full recyclability.

Beyond transesterification-based vitrimers, alternative dynamic chemistries have also been adapted for photocurable 3D printing with enhanced recyclability. Menasce^96^ et al. developed recyclable silicone vitrimers fabricated via DIW combined with photocuring, employing dioxaborolane groups as dynamic cross-links. The boronic ester exchange mechanism operates with exceptionally low activation energy (8.5 kJ/mol), enabling room-temperature reprocessability a significant advantage over transesterification-based systems that typically require elevated temperatures for network rearrangement. Silica nanofillers served dual functions: tuning ink rheology for DIW processability and mechanically reinforcing the photocured network. The printed vitrimer parts could be amended at room temperature through simple contact under gentle pressure, and recycled materials retained mechanical properties comparable to pristine samples. This approach extends the vitrimer toolkit for recyclable photopolymer 3D printing to elastomeric materials with complex geometries unattainable by vat-based methods (Figure 6a–c).

**Figure 6:**
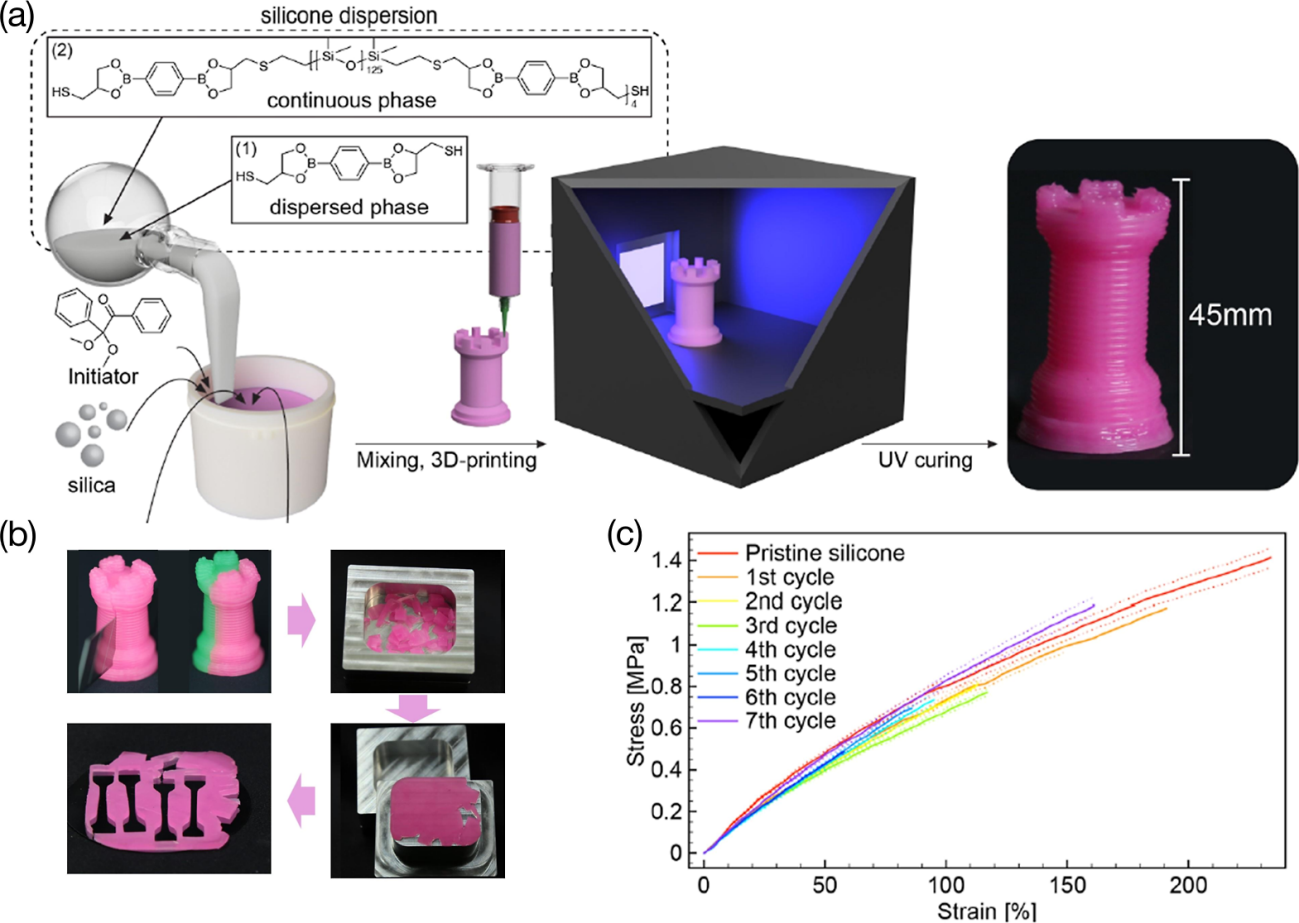
(a) Schematics displaying the mixing, 3D printing, and UV curing of inks containing silicone prepolymer, thiol-ended dioxaborolane, siloxane cross-linker, silica nanofillers, photoinitiator, and photoabsorber. (b) Photographs depicting the amendability of printed silicone vitrimers, and photographs displaying a reprocessing cycle of the silicone vitrimer at room temperature. A fragmented sample is compressed in a mold to generate a silicone monolith. Dogbone specimens are extracted from the monolith for mechanical testing. (c) Tensile stress–strain curves obtained from vitrimer samples subjected to various reprocessing cycles. Adopted with permission from ref 96. Copyright 2021, American Chemical Society.

Conventional vitrimer systems typically require catalysts to achieve practical exchange reaction rates, which may introduce concerns regarding long-term stability and biocompatibility. Cui^97^ et al. addressed this limitation by developing catalyst-free exchangeable networks exploiting the electron-withdrawing effect of β-positioned carbonyl groups to facilitate transesterification between hydroxyl and ester moieties without additional catalysts. The resulting photocurable resin can be directly UV-cured into crosslinked networks that undergo topological rearrangement and dissociation through transesterification. Dissociation products serve as feedstock for renewed DLP 3D printing. Beyond recyclability, these materials exhibit tailorable mechanical properties, elastomeric behavior, and shape-memory capabilities, opening new avenues for intelligent 3D printing applications.

### 3.4 Recyclable Networks Based on Noncovalent Interactions

Concovalent interactions including hydrogen bonding, metal coordination, and ionic association offer an alternative paradigm for constructing recyclable photopolymer networks. The relatively low dissociation energies of noncovalent bonds enable network disruption and reformation under mild conditions, potentially reducing the energy requirements for recycling compared to systems relying on covalent bond exchange. While networks crosslinked solely through noncovalent interactions typically exhibit limited mechanical properties, strategic combination with covalent networks can achieve synergistic enhancement of both performance and recyclability.

Huang^37^ and colleagues developed a biobased carboxyl-terminated unsaturated polyester (PHI) from itaconic acid and biomass-derived 1,6-hexanediol for solvent-based photopolymerization (Figure 7a,b). The pendant double bonds in PHI exhibit higher mobility than backbone double bonds in traditional unsaturated polyesters, conferring superior photoreactivity. Terminal carboxyl groups coordinate with metal ions such as ZnCl_2_ to form noncovalent crosslinks that synergistically construct a dual network with covalent crosslinks from pendant double bond polymerization. This architecture significantly enhances mechanical properties while enabling dynamic network rearrangement through ZnCl_2_-catalyzed reversible transesterification. Crushed 3D printed products can be reprocessed via hot-pressing with 70% strength recovery, offering a sustainable alternative to conventional acrylate prepolymers.

**Figure 7:**
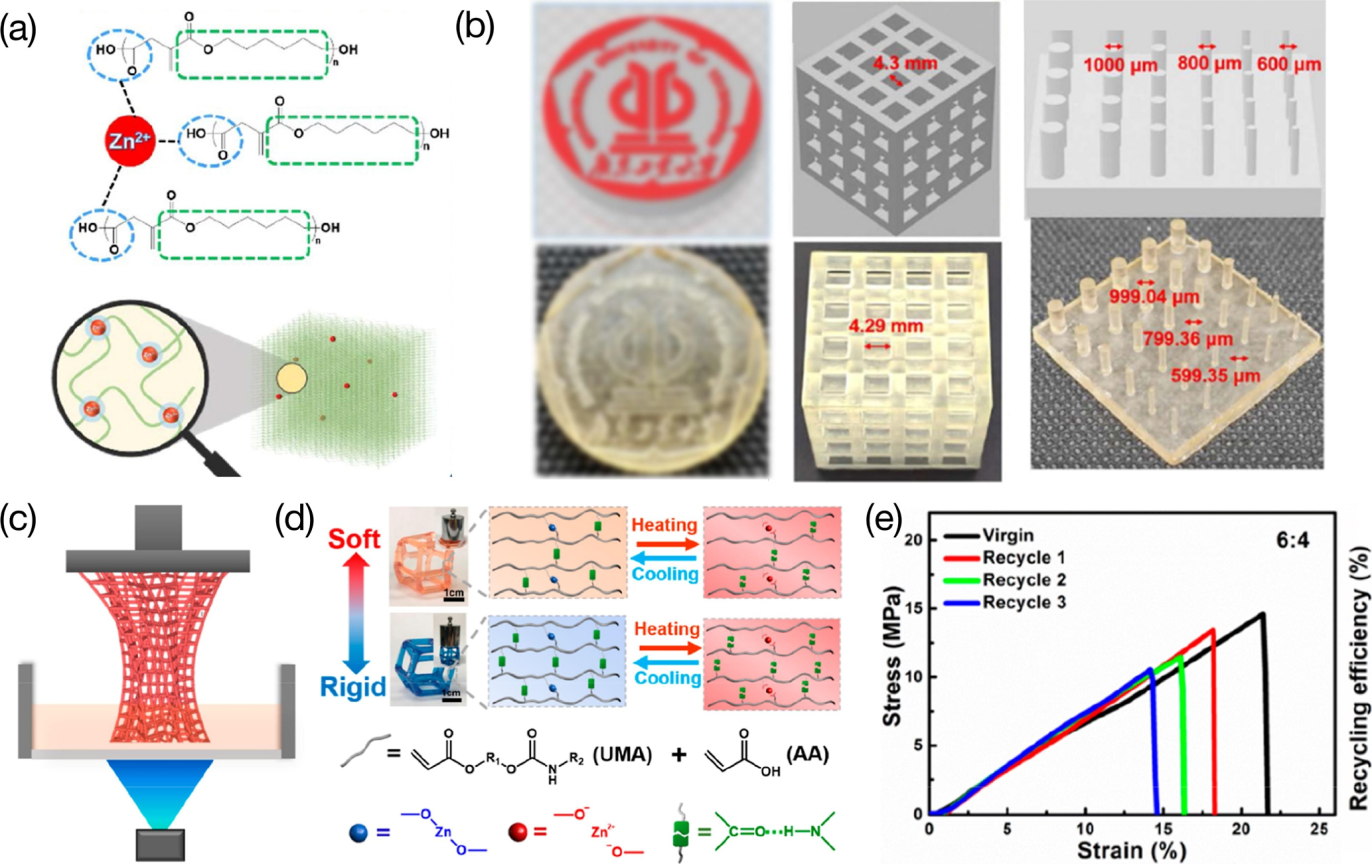
(a) Schematic illustration of coordination interaction between ZnCl_2_ and biobased carboxyl-terminated unsaturated polyester (PHI). (b) 3D printed objects of university badge, grid cube and different sized cylinders from PHI_1.2_–Zn_2_ wt % resin. Adopted with permission from ref 37. Copyright 2024, Elsevier B.V.; (c) scheme of the DLP printing process. (d) Dynamic polymer networks crosslinked by reversible ionic bonding and hydrogen bonding. (e) Representative stress–strain curves of the samples with urethane monoacrylate/acrylic acid (UMA/AA) = 6:4 after multiple cycles of recycling. Adopted with permission from ref 33. Copyright 2021, American Chemical Society.

Circumventing traditional chemical crosslinking entirely, Zhao^33^ et al. reported printable resins for fabricating self-healing and recyclable polymers with tunable properties ranging from soft elastomers to rigid plastics (Figure 7c–e). These resins contain urethane monoacrylate and acrylic acid as monofunctional monomers with zinc dimethacrylate as the crosslinker. Photopolymerization generates solid structures crosslinked through ionic bonding and hydrogen bonding between monomer units rather than covalent crosslinks. The dynamic nature of these interactions enables damaged printed objects to undergo self-repair and recycling, restoring original structure and mechanical performance.

### 3.5 Comparative Analysis

The preceding sections have presented diverse strategies for achieving recyclability in 3D printed photopolymers. To facilitate rational material selection for specific applications, this section provides a comparative analysis of the printing method, recycling efficiency, and processing conditions, of each approach (Table 2). Chemical recycling via depolymerization offers the highest theoretical recovery fidelity, as regenerated monomers are chemically equivalent to virgin feedstock. This approach is particularly advantageous for applications demanding consistent material properties across multiple use cycles. However, depolymerization typically requires specific triggering conditions (elevated temperature, chemical agents, or light exposure) and may involve longer processing times compared to thermo-mechanical approaches. The ceiling temperature of the polymer system imposes fundamental constraints on both the service temperature range and the conditions required for depolymerization. Thermo-mechanical reprocessing via dynamic bond exchange offers processing simplicity, as recycling proceeds through conventional thermoplastic techniques without requiring depolymerization or dissolution steps. This approach is well-suited for applications where moderate property retention across cycles is acceptable and processing infrastructure for hot pressing or molding is available. However, repeated thermal cycling may induce oxidative degradation, and the requirement for elevated temperatures and pressures increases energy consumption. Noncovalent interaction-based networks enable recycling under the mildest conditions, minimizing energy requirements and thermal degradation risks. These systems are particularly attractive for applications requiring repeated reshape cycles or self-healing functionality. The primary limitation lies in their generally lower mechanical properties compared to covalently crosslinked systems, although hybrid architectures combining noncovalent and covalent crosslinks can partially address this trade-off.

**Table 2:** Summary of Different 3D Printing Methods for Recyclable Photopolymer Systems^a^

refs	polymer class	printing method	recycling mechanism	processing conditions	recycling efficiency	cycle number	mechanical properties	property retention
Dove et al.^92^	polythioether	DLP	depolymerization	heat; acid catalyst	>90% monomer	multiple	*E* = 2.5 GPa	>95%
Xie et al.^80^	polydithioacetal	SLA	depolymerization	acid hydrolysis, RT	∼100% monomer	3	σ = 45 MPa	∼90%
Yue et al.^34^	polyester	DLP	depolymerization	mild acid, 60 °C	>85% monomer	multiple	σ = 35–50 MPa	>90%
Wang et al.^2^	polyacrylate	DLP	depolymerization	aqueous acid, RT	>90% monomer	multiple	σ = 40–55 MPa	>92%
Zhang et al.^93^	acrylate vitrimer	DLP	transesterification	180 °C, Zn catalyst	∼100% (reshaping)	>5	σ = 25–40 MPa, *E* = 1.2 GPa	∼85%
Li et al.^91^	acrylate vitrimer	DLP	transesterification	160–180 °C, catalyst	∼100% (reprocessing)	3–5	σ = 30–45 MPa	∼80–90%
Gao et al.^94^	acrylate vitrimer	SLA	transesterification	180 °C hot-pressing	∼100% (reshaping)	multiple	σ = 40 MPa, *E* = 871 MPa	>85%
Jehl et al.^95^	polybenzoxazine vitrimer	DIW	transesterification	thermal post-cure	∼100% (grinding reuse)	multiple	*T*_α_ = 105 °C, *E* = 810 MPa	>80%
Menasce et al.^96^	silicone vitrimer	DIW	boronic ester exchange	RT	∼100% (self-healing)	multiple	NA	NA
Cui et al.^97^	acrylate vitrimer	DLP	transesterification	200 °C, no catalyst	∼100% (reshaping)	>3	σ = 35–50 MPa	∼85%
Huang et al.^37^	unsaturated polyester	DLP	ionic interactions	water dissolution, RT	>95% (dissolution)	multiple	σ = 15–25 MPa	>90%
Zhao et al.^33^	polyurethane-acrylate	DLP	ionic and H-bond	solvent treatment	>90% (reprocessing)	multiple	σ = 20–35 MPa	>85%

a Abbreviations: σ = tensile strength; *E* = Young’s modulus; *T*_α_ = alpha transition temperature; RT = room temperature; H-bond = hydrogen bond.

## 4 Applications Based on 3D Printing Using Photopolymers

### 4.1 Soft Robots

Soft robots represent a class of continuum actuators constructed from flexible intelligent materials. Their defining characteristic lies in interacting with environments and executing tasks through distributed elastic deformation of their intrinsic structure rather than via traditional rigid joint movements. Currently, soft robots can be engineered to leverage external environmental stimuli such as temperature and pH as driving sources, enabling controlled shape transformations and movements.^98–101^

For example, Zhou et al.^102^ have developed a novel ultraviolet UV-curable polyurethane acrylate prepolymer based on castor oil (CO)-based disulfide bond (S–S)-containing UV-curable polyurethane acrylate prepolymer (COPUA-SS). Owing to the presence of dynamic disulfide bonds and urea bonds, which undergo reversible exchange reactions at elevated temperatures, objects formed by DLP 3D printing of this resin exhibit exceptional self-healing capabilities, recyclability (Figure 8a) with a tensile modulus recovery rate as high as 168.4% (Figure 8b), plastic deformation capacity, and programmable shape memory properties. A palm-shaped structure fabricated via DLP 3D printing not only achieves temporary shape fixation and recovery at 100 ^o^C with a shape fixation rate exceeding 87% but also can be reshaped into new permanent configurations such as a helical form at 140 °C while maintaining subsequent shape memory responsiveness (Figure 8c,d). These attributes establish a solid foundation for its application in fields including flexible robotic actuators and deformable structures. Rossegger et al.^54^ designed a polymer resin featuring a dynamic covalent adaptive network and successfully fabricated structured devices from this material using bottom-up digital light processing technology. Once photopolymerized these devices demonstrate rapid thermally activated rearrangement of their network topology through stress relaxation experiments enabled by the dynamic thiol-click chemistry. This rapid response capability facilitates controllable macroscopic deformation. Leveraging this property the team demonstrated a gripper rapidly capturing an object upon thermal activation. The combination of fast response speed and design flexibility renders these networks highly attractive candidates for manufacturing customized active materials in soft actuators and robotics (Figure 8e,f).

**Figure 8:**
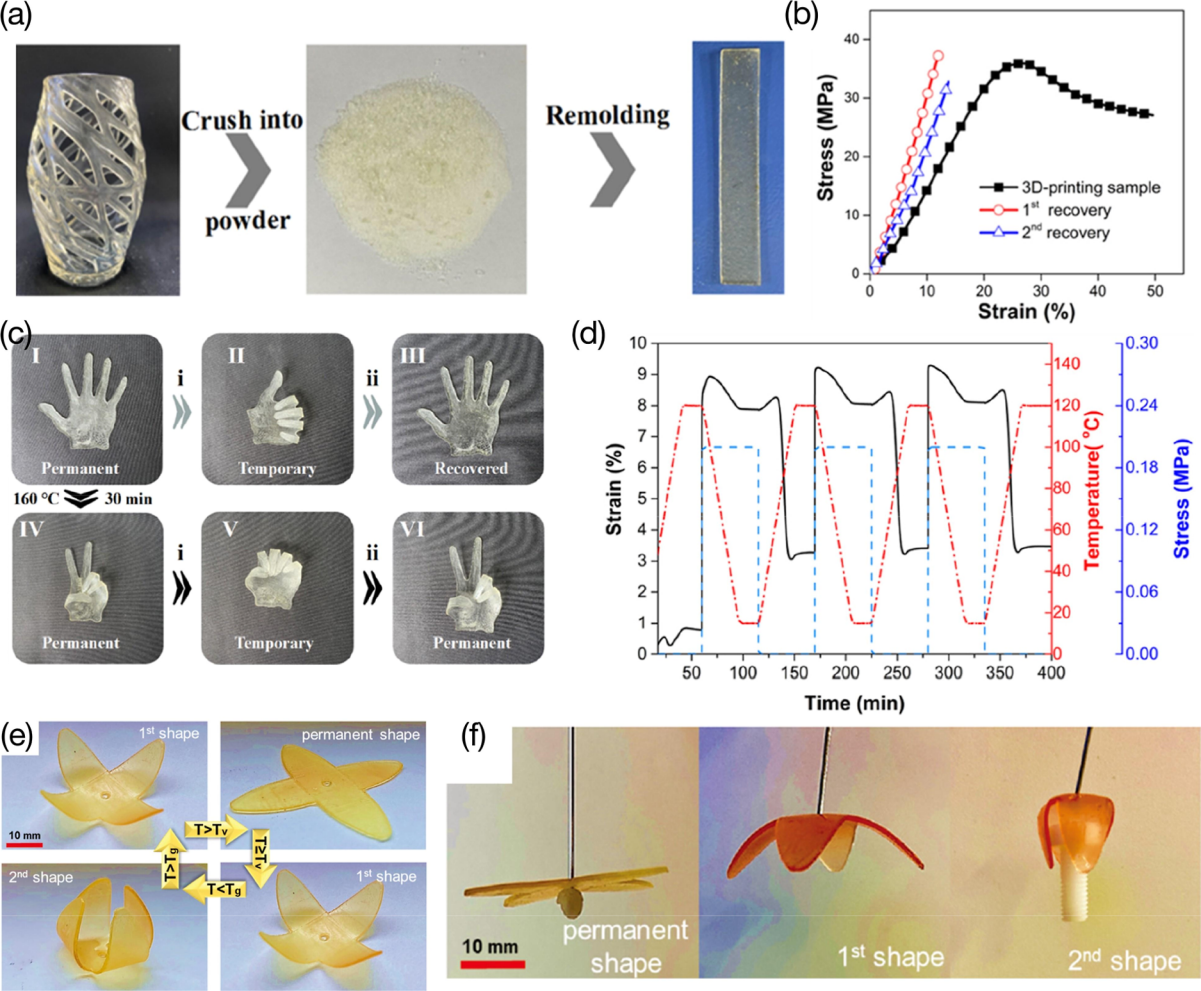
(a) Recycling process of the printed flower basket model. (b) Stress–strain curves. (c) Visual demonstration of temporary and permanent shape changing performance where (i) denotes the shape changing process at 120 °C and shape fixing process in cold water and (ii) denotes the shape recovery process at 120 °C. (d) Consecutive dual-shape memory cycles of the castor oil (CO)-based disulfide bond (S–S)-containing UV-curable polyurethane acrylate prepolymer (COPUA-SS) sample. Adopted with permission from ref 102. Copyright 2025, Elsevier B.V.; (e) photographs monitoring the triple-shape memory of DLP printed samples. (f) DLP printed gripper showing the potential of this new class of material for the customized production of structural and fast acting devices. Adopted with permission from ref 54. Copyright 2021, Royal Society of Chemistry.

### 4.2 Wearable Devices

As a crucial interface connecting humans with intelligent environments, wearable devices rely on sensors as their core components, which can convert external physical stimuli such as mechanical force, thermal signals, and chemical signals into quantifiable electrical signals.^103–105^ These sensors exhibit substantial application potential in cutting-edge fields including personalized physiological monitoring human motion detection and intelligent human-machine interfaces. Recently, 3D printing has offered unique solutions for high-performance flexible sensor manufacturing owing to its excellent compatibility with soft materials digital freedom for fabricating complex 3D geometries and rapid design-to-manufacture iteration capabilities. As exemplified by one study researchers successfully fabricated a biomimetic cuttlebone-inspired robotic sensing device using DLP 3D printing (Figure 9a,b).^106^ The printed composite demonstrated not only significant piezoelectric response and mechanical load-bearing capacity but also integrated exceptional self-healing and chemical recyclability highlighting the advantage of 3D printing for structure–function integration. Similarly intelligent arrayed armor and derived knee pads developed via 3D printing enable real-time precise monitoring of force displacement and load magnitude during wearer establishing a foundation for practical wearable sensing devices Figure 9c,d.

**Figure 9:**
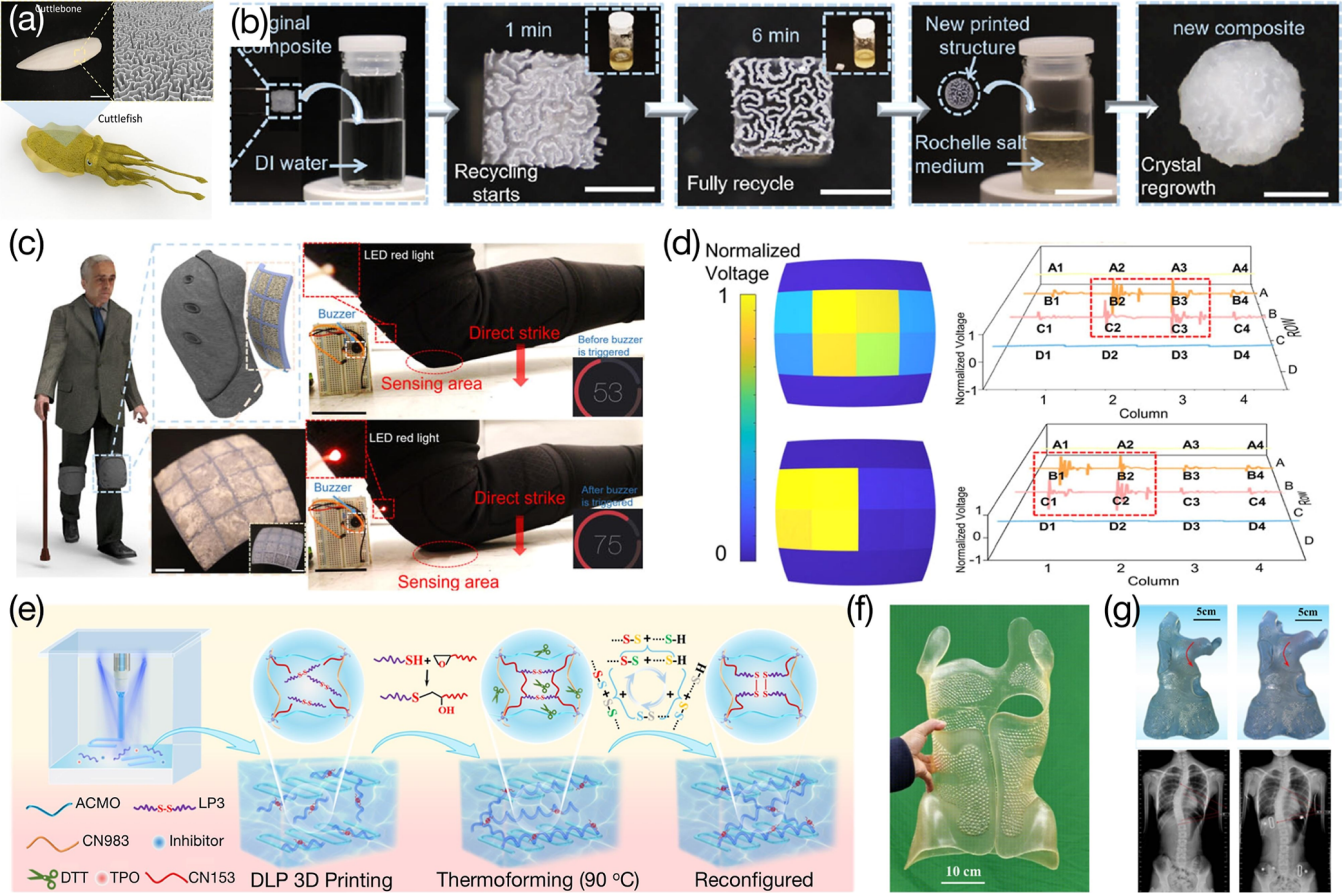
(a) Schematic diagram of bio-inspired 3D printed cuttlefish bone structure and RS crystal growth process. (b) The photos of the process for 3D printed rochelle salt cuttlebone composite (RSC) recycling. (c) Schematic diagrams and images of knee pad (scale bar, 10 mm), as well as an alarm detection test for knee pad (scale bar, 30 mm). (d) MATLAB element block distribution and the voltage waveforms of output voltage obtained from smart knee protector fall test. Adopted with permission from ref 106. Copyright 2023, Springer Nature; (e) mechanism diagram of the orthosis with dynamicity prepared via 3D printing. (f) The optical image of the 3D printed orthosis. (g) Adjustment of the angle to suit the change in physical condition, with the angle of scoliosis measured via X-ray. Adopted with permission from ref 107. Copyright 2024, Royal Society of Chemistry and the Chinese Chemical Society.

He and colleagues^107^ have developed a rigid yet reconfigurable, recyclable, and repairable polymer that can be fabricated into wearable orthoses via DLP 3D printing (Figure 9e). This engineered polymer exhibits high mechanical strength, with a Young’s modulus of 363.2 MPa and a maximum elongation of 146.3%, along with excellent dynamic properties including the ability to be repeatedly processed at 100 °C, recycled upon dissolution in thiol-containing solvents, and repaired through heating. Wearable orthoses manufactured by 3D printing with this polymer can adapt to the patient’s body through continuous reconfiguration, thereby enhancing their effectiveness in treating adolescent idiopathic scoliosis (Figure 9f,g). Furthermore, these orthoses can be recycled after use, offering the potential to significantly reduce medical waste.

### 4.3 Bioelectronic Devices

Bioelectronic devices characterized by flexibility bendability and biocompatibility find widespread application in areas such as human-machine interaction biomedical applications and biosignal detection. Significantly the increasing sophistication of contemporary bioelectronics stems from considerable attention directed towards further device miniaturization enhancing performance through multimodal functionalities developing soft and stretchable electronics and customizing devices for improved compatibility and personalized point-of-care applications.^108–110^ These advances have enabled the broad utilization of various materials and composites meticulously engineered to achieve diverse structures properties and functionalities. Recent advancements in 3D printing have opened new avenues for the fabrication of bioelectronic devices.

Smaldone et al.^111^ synthesized five resin formulations derived from bio-based vanillin successfully printing these materials using digital light processing technology. The presence of dynamic covalent crosslinks within these thermosets enables them to be reprocessed under high pressure using a thermal press at temperatures above their glass transition. This reprocessability effectively supports the development of closed-loop manufacturing pathways for bioelectronic devices spanning initial fabrication to end-of-life recycling. However, the flexible substrate materials widely used in current bioelectronics face significant limitations regarding recyclability and reprocessability. This challenge directly contributes to the generation of substantial non-degradable electronic waste, leading not only to resource depletion but also posing potential threats to ecosystems and organismal health through the leaching of toxic components (Figure 10a,b). To address this issue, Tao et al.^112^ developed a recyclable polyurethane (PU) copolymer system based on dynamic covalent interactions (Figure 10c). Through molecular design, reversible boron–nitrogen coordination bonds were incorporated into the polymer network. Under specific conditions, this material undergoes controlled decomposition (Figure 10d). It can reversibly dissociate into small molecular fragments containing boric acid and hydroxyl groups via the cleavage and reformation of boron–nitrogen bonds, or undergo bond exchange reactions through a synergistic pathway enabling dynamic rearrangement of the network topology. As a flexible substrate, this recyclable PU copolymer has been effectively applied in various wearable and implantable bioelectronic devices. It ensures good biocompatibility while enabling stable, high-quality electrophysiological signal recording and neural stimulation functions. Critically, the system establishes a closed-loop sustainable recycling model. Small molecule monomers or oligomers obtained through chemical recycling processes can be repolymerized into the PU copolymer. This regenerated material can then be used to reconstruct a range of bioelectronic devices, with mechanical properties such as elastic modulus and elongation at break maintaining their original levels even after multiple recycling cycles. This closed-loop approach not only addresses the key challenge of recycling medical electronic waste but also paves the way for developing sustainable flexible bioelectronics suitable for healthcare applications (Figure 10e,f).

**Figure 10:**
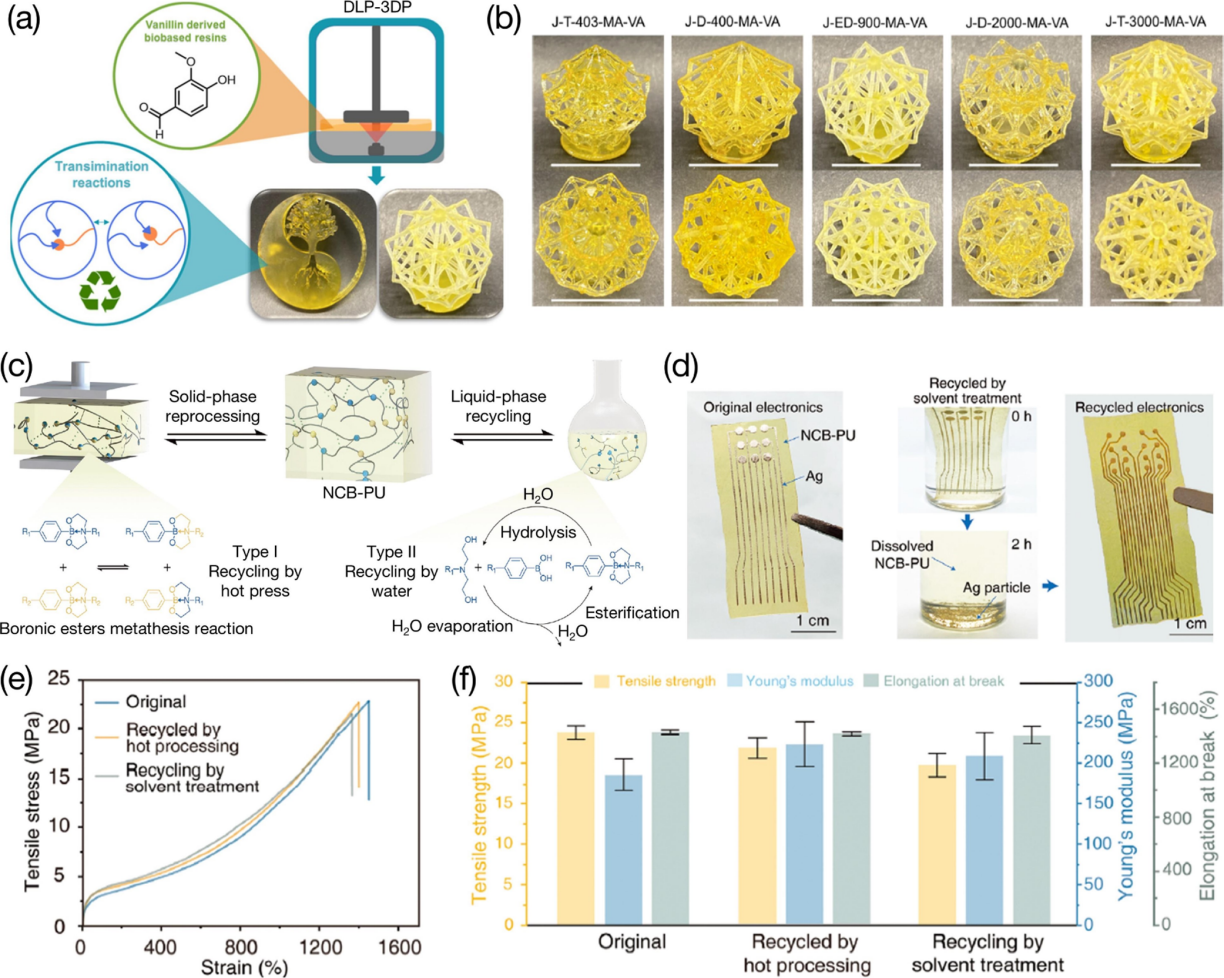
(a) Schematic diagram of 3D printed recyclable biobased photopolymer resin. (b) Complex structures printed with the five different Vanillin–Jeffamine formulations. Adopted with permission from ref 111. Copyright 2022 American Chemical Society. (c) Schematic diagram of the proposed mechanism for recyclable nitrogen-coordinating cyclic boronic diester-polyurethane (NCB-PU) vitrimers and reversible reaction of NCB linkages in PU networks induced by water or boronic esters metathesis. (d) Image of the wearable electronics equipped with nine autonomous channels and image of the solvent-induced recovery of NCB-PU-based bioelectronics reaction. (e) The stress–strain curves of recycled NCB-PU sample. (f) Summary of mechanical attributes of the recycled NCB-PU vitrimers by hot processing and solvent treatment. Adopted with permission from ref 112. Copyright 2024, Wiley-VCH.

## 5 Summary and Outlook

The development of recyclable photopolymers for 3D printing is vital for overcoming the recycling challenges inherent in thermosets used within this technology and for achieving closed-loop material cycles and resource sustainability in additive manufacturing. This review examines significant progress in recyclable photopolymers for 3D printing, highlighting their pivotal role in advancing sustainable additive manufacturing. It details the compatibility of mainstream photopolymerization-based techniques with these materials, explores core material design strategies incorporating dynamic covalent bonds and noncovalent interactions, and elucidates fundamental recycling mechanisms encompassing chemical recycling and thermomechanical reprocessing. Building on this foundation, the discussion extends to performance modulation approaches and the promising application potential of recyclable photopolymers in emerging fields such as soft robotics, wearable devices, and bioelectronic interfaces. These developments collectively underscore the substantial potential of recyclable photopolymers to mitigate the environmental impact of 3D printing by enabling closed-loop material lifecycles while simultaneously providing a versatile platform for fabricating advanced functional devices. To fully realize the transformative potential of this technology and address existing limitations, several key research directions merit focused investigation:

1. Enhancing chemical recycling universality. Future efforts should focus on broadening the applicability of chemical recycling to diverse photopolymer chemistries. Optimizing the incorporation of tailored dynamic motifs, such as exchangeable bonds responsive to specific stimuli, is crucial to enable efficient depolymerization across resin classes while minimizing mechanical property degradation over successive cycles. Developing catalyst-free, closed-loop photoprinting platforms achieving near-zero waste remains a primary objective.^113,114^

2. Optimizing thermo-mechanical processes. Refining thermo-mechanical recycling necessitates in-depth investigation into processing parameters like temperature profiles, pressure regimes, and particle morphology control.^115,116^ Understanding the interplay between these parameters and the resulting recycled material performance is vital to mitigate property deterioration and energy consumption, enhancing the viability of this physical recycling route.
3. Bridging the robustness-recyclability gap. Overcoming the inherent trade-off between dynamic bond reversibility and mechanical robustness requires innovative material design.^117^ Exploring novel dynamic covalent chemistries with higher bond energies, such as reversible Si–O–Si or boroxine networks, presents a promising pathway to develop stronger, high-performance photopolymers without compromising recyclability, particularly for demanding structural applications.^118^
4. Integrating multi-mechanism recycling. Developing photopolymer systems that synergistically combine multiple recycling mechanisms e.g., concurrent chemical and thermo-mechanical pathways, or covalent/noncovalent interplay within a single material platform could unlock superior recyclability, enhanced functionality e.g., self-healing, and broader application scope, moving beyond single-mechanism limitations.^119^
5. Exploring convergence with 4D printing functionality. 4D printing, which introduces time as the fourth dimension through stimuli-responsive shape or property transformations, represents an emerging Frontier that naturally intersects with recyclable photopolymer development.^120,121^ Dynamic covalent networks, such as vitrimers, exhibit programmable shape-memory behavior due to their ability to fix temporary shapes below the topology freezing temperature and recover permanent shapes upon reheating. Similarly, networks incorporating noncovalent interactions can undergo reversible shape changes in response to external stimuli such as temperature, humidity, or pH. The integration of recyclability with 4D functionality offers exciting opportunities for developing next-generation smart materials that are simultaneously responsive, reprogrammable, and sustainable. Future research should explore how to maximize both shape-programming fidelity and recycling efficiency within a single material platform, potentially enabling applications in adaptive soft robotics, deployable structures, and personalized biomedical devices that can be reconfigured during use and responsibly recycled at end-of-life.

Overall, the collective progress in novel dynamic chemistries, sophisticated network design, and application-driven development marks a pivotal step towards unlocking the full potential of sustainable and functional 3D printing, paving the way for its integration into circular manufacturing ecosystems.
